# Brain injury drives optic glioma formation through neuron-glia signaling

**DOI:** 10.1186/s40478-024-01735-w

**Published:** 2024-02-02

**Authors:** Jit Chatterjee, Joshua P. Koleske, Astoria Chao, Andrew D. Sauerbeck, Ji-Kang Chen, Xuanhe Qi, Megan Ouyang, Lucy G. Boggs, Rujuta Idate, Lara Isabel Marco Y Marquez, Terrence T. Kummer, David H. Gutmann

**Affiliations:** Department of Neurology, Washington University School of Medicine, 660 S. Euclid Avenue, Box 8111, St. Louis, MO 63110 USA

**Keywords:** Optic glioma, Brain tumor, Cytokine, Traumatic brain injury, Optic nerve crush, Microglia, Tumor microenvironment, Tumorigenesis

## Abstract

Tissue injury and tumorigenesis share many cellular and molecular features, including immune cell (T cells, monocytes) infiltration and inflammatory factor (cytokines, chemokines) elaboration. Their common pathobiology raises the intriguing possibility that brain injury could create a tissue microenvironment permissive for tumor formation. Leveraging several murine models of the Neurofibromatosis type 1 (NF1) cancer predisposition syndrome and two experimental methods of brain injury, we demonstrate that both optic nerve crush and diffuse traumatic brain injury induce optic glioma (OPG) formation in mice harboring *Nf1*-deficient preneoplastic progenitors. We further elucidate the underlying molecular and cellular mechanisms, whereby glutamate released from damaged neurons stimulates IL-1β release by oligodendrocytes to induce microglia expression of Ccl5, a growth factor critical for *Nf1*-OPG formation. Interruption of this cellular circuit using glutamate receptor, IL-1β or Ccl5 inhibitors abrogates injury-induced glioma progression, thus establishing a causative relationship between injury and tumorigenesis.

**Supplementary Information:**

The online version contains supplementary material available at 10.1186/s40478-024-01735-w.

## Introduction

The correlation between inflammation and cancer was first proposed by Rudolf Virchow in the nineteenth century based on the finding that cancers often originate at sites of chronic inflammation and that inflammatory cells are frequently abundant in tumors [8]. While inflammation can be triggered by various factors, including infection or tissue damage (short-lived), inflammatory factors can induce cellular proliferation and prolong cell survival through interactions established by mutations in growth regulatory genes [39]. For example, activation of the RET proto-oncogene, which is sufficient and necessary to induce papillary thyroid cancer, induces an inflammatory transcriptional program, resulting in the elaboration of interleukins, inflammatory cytokines, and chemokines [10]. Similarly, expression of oncogenic *KRAS* and *MYC* genes (Myc proto-oncogene) instructs the production of inflammatory cytokines and chemokines that maintain key aspects of tumor biology [3, 27, 52, 54].

In the setting of the Neurofibromatosis type 1 (NF1) cancer predisposition syndrome, both central (optic glioma) and peripheral (neurofibroma) nervous system low-grade tumor development and growth are dependent upon cytokines and chemokines produced by non-neoplastic immune cells in the cancer microenvironment. Leveraging *Nf1* genetically engineered mouse (GEM) models of plexiform neurofibromas [38], mast cells and T cells drive tumor formation and growth through the production of Kit ligand [61] and cytokines, like Cxcl10 [23], respectively, as well as through sustained expression of the periostin (Postn) injury response gene and increased NFkB signaling [22, 33]. Analogous to neurofibroma development, nerve injury attracts immune cells, such as macrophages and mast cells [14], to establish an inflammatory microenvironment. In this regard, the induction of cytokines and growth factors by neoplastic Schwann cells in neurofibromas resembles that observed in injury-induced Schwann cells [22]. Likewise, skin injury also accelerates the development and growth of cutaneous neurofibromas in *Nf1*-mutant mice, associated with increased Ccl2 and Ccl5 expression [48], while partial sciatic nerve transection induces the formation of neurofibromas in *Nf1*-mutant mice at the site of the injury [49].

In the central nervous system (CNS), mouse *Nf1* optic gliomas require the elaboration of Ccl5 from tumor-associated monocytes (TAMs) [53]. Previous studies from our laboratory have characterized a “neuron-immune-cancer cell” axis, in which neurons elaborate midkine in an activity-dependent manner [5], which stimulates T cell Ccl4 production to induce TAM (Tmem119^+^/Iba1^+^/CD45^low^/CD11b^+^ microglia [19, 46]) Ccl5-mediated support of glioma growth [28]. As such, Ccl5 is sufficient to increase the survival of optic glioma tumor cells, and its inhibition, either using neutralizing antibodies, Ccl5 receptor inhibitors, or genetic knockdown, abrogates tumor growth. Since Ccl5 is commonly induced by traumatic brain injury in both experimental rodent models [25, 32] and patients [1], we sought to explore the intersection between CNS injury and optic gliomagenesis using authenticated preclinical *Nf1*-mutant GEM strains. Capitalizing on the high tumor penetrance, stereotypic tumor location (prechiasmatic optic nerve and chiasm), and well-defined temporal course of optic glioma development in GEM [19, 55], we employed several different *Nf1*-mutant mouse lines and two complementary experimental approaches to demonstrate that brain injury establishes a supportive microenvironment sufficient for optic glioma formation in mice with progenitor cell *Nf1* loss. Moreover, we elucidate a new neuron-glia paracrine circuit, in which neuronal glutamate stimulates oligodendrocyte IL-1β production to result in TAM Ccl5-mediated tumor growth.

## Materials and methods

### Mice

All experiments were performed under an active and approved Animal Studies Committee protocol at Washington University School of Medicine (Institutional Animal Care and Use Committee). Mice were maintained on a 12-h light/dark cycle in a barrier facility with ad libitum access to food and water. Several lines of mice were used for these experiments, including *Nf1*^*flox/flox*^; hGFAP-Cre mice (*Nf1*^+*/*+^ mice with somatic *Nf1* gene inactivation in neuroglial progenitors [7]), *Nf1*^flox/mut^; GFAP-Cre (*Nf1*^OPG^) mice (*Nf1*^+*/−*^ mice with somatic *Nf1* gene inactivation in neuroglial progenitors at E16.5), *Nf1*^+*/*−^ mice (neomycin sequence insertion within exon 31 of the murine *Nf1* gene [11]), and *Nf1*^flox/R1809C^; GFAP-Cre (*Nf1*^R1809C^) mice (*Nf1*^+*/R1809C*^ mice with somatic *Nf1* gene inactivation in neuroglial progenitors at E16.5 [5]). Littermate *Nf1*^flox/flox^ mice were used as controls. Mice of both sexes were randomly assigned to all experimental groups without bias, and the investigators were blinded until the final data analysis. In accordance with Washington University IACUC guidelines, animals with compromised motion/eating habits or an unhealthy appearance were euthanized; however, no mice were euthanized due to tumor burden or as a result of the treatments performed in this study.

### Optic nerve crush

Mice were anesthetized by intraperitoneal injection of a sterile mixture of ketamine (100 mg/kg) and xylazine (10 mg/kg). After a surgical level of anesthesia was achieved, a small incision was made in the conjunctiva with spring scissors, beginning inferior to the globe and around the eye temporally, according to published protocols [15]. The exposed optic nerve was grasped approximately 1–3 mm distal to the globe for 10 s, with only pressure from the action of the self-clamping forceps to press on the nerve. After 10 s, the optic nerve was released and the forceps removed, allowing the eye to rotate back into place. Optic nerve crush was performed both unilaterally and bilaterally in separate experiments. At least 5 mice per group were used in all experiments.

### Traumatic brain injury

Traumatic brain injuries were performed using the modCHIMERA model as previously described [51], with sole exception being an impact energy of 0.77 ± 0.009 Joules (J). In brief, prior to initiation of the brain injury mice were anesthetized with 5% isoflurane for 2 min and 15 s followed by maintenance at 2.5% for the remainder of the experiment. The entire experiment took 5–7 min per animal. After the initial induction of anesthesia, a custom helmet was placed on the animals’ head and the animal was positioned such that the impact would occur on the midline of the skull 4 mm posterior to the lateral canthus of the eye. Immediately following the impact, eye lubricant was applied to the eyes, the animal was monitored for effective breathing, and recovery occurred in a warming box until the animal fully regained ambulatory function. At least 5 mice per group were used in all experiments.

### Optic nerve volume determinations

Isolated optic nerves were photographed using a Leica S9D with a Flexicam C3 camera, and their volumes calculated as previously described [16]. Using ImageJ (version 10.1), four diameter measurements were taken to estimate the thickness of each optic nerve, beginning at the chiasm (D_0_), at 150 µm (D_150_), 300 µm (D_300_), and 450 µm (D_450_) anterior to the chiasm. The volumes for regions 1, 2, and 3 at the three 150 μm high-truncated cones were merged using the diameter (D0, D150, D300, and D450) values from each optic nerve measurement. The following equation was used to calculate optic nerve volumes: V_1_ = 1/12 *π*h (D_0_^2^ + D_0_D_150_ + D_150_^2^).

### Immunohistochemistry and immunocytochemistry

Mice were euthanized and transcardially perfused with Ringers solution, followed by 4% paraformaldehyde (PFA) fixation. Fixed tissue was processed for paraffin embedding. Serial 4-μm paraffin sections of the optic nerve were immunostained with appropriate primary and secondary antibodies (Additional file 1: Table S1) and developed using the Vectastain ABC kit (Vector Laboratories, PK4000). For immunocytochemistry, sections of optic nerve were immunostained with appropriate primary and secondary Alexa-fluor-conjugated antibodies (Additional file 1: Table S1). Images of the optic nerve chiasm were acquired using Zeiss AxioScan-Z1 and Leica ICC50W microscopes with LAS EZ software or a Leica DMi8 fluorescent microscope with LAS X software.

### Microglia isolation

Microglia were isolated from D-PBS-perfused mouse brains. Single cell suspensions were generated with the multi-tissue dissociation kit (Miltenyi Biochemicals) using established protocols [16]. The resulting cells were maintained in minimal essential medium supplemented with 1 mM L-glutamine, 1 mM sodium pyruvate, 0.6% D-glucose, 1 ng/ml GM-CSF, 100 μg/ml P/S, and 10% fetal bovine serum (FBS). At 14 days in vitro, microglia were mechanically dissociated from the astrocyte layer by gentle shaking (200 g, 5 h, 37 °C). 5 × 10^5^ microglia per replicate were used for the IL-1β treatment experiments.

### Oligodendrocyte isolation

Mixed glial cultures were generated from 1 to 2-day-old mice pups as described previously [17]. Briefly, the cerebra of mice pups were dissected, minced, and digested at 37 °C using a neural tissue dissociation kit (Miltenyi Biotec, catalog #130-092-628) to generate a single-cell suspension. Cells were plated into 75-cm^2^ flasks and grown in DMEM with 10% fetal bovine serum (FBS) at 37 °C and 5% CO_2_ for the next 10 days. After 6–8 days, oligodendrocyte precursor cells (OPCs) can be visualized growing upon a mesh of astrocytes. On the 10th day, OPCs were purified from mixed glial cells by shaking. Cells were shaken initially for 1 h at 200 rpm to remove microglia, refed, and shaken again for 18–20 h at 37 °C at 200 rpm. OPCs were collected by centrifugation at 10 min at 100 × *g*. OPCs were then cultured in oligodendrocyte medium (Sciencell, catalog #1621). PDGF-AA (Sigma) was added at 10 ng/ml to Sato medium for allow for oligodendrocyte growth.

### RNA extraction and real-time PCR

Using the NucleoSpin® RNA Plus kit (Takara-740984.205), total RNA was isolated from the optic nerves of mice according to the manufacturer’s instructions. Isolated RNA was then reverse transcribed into cDNA using the Applied Biosystems High-Capacity cDNA Reverse Transcription Kit (#4374967) as per the manufacturer’s instructions. Real-time quantitative PCR (qPCR) was performed by TaqMan gene expression (Additional file 1: Table S2). ΔΔCT values were calculated using *Gapdh* as an internal control. All reactions were performed using the QuantStudio 3 system (Applied Biosystems). For each experiment, two mice were used per sample and 3 or 4 samples were included in each experiment.

### Western blotting

Total protein was extracted from cells and snap frozen tissues using RIPA buffer supplemented with a protease inhibitor cocktail and quantified using the Pierce BCA protein assay kit (Fisher scientific, PI23225). 40 µg of total protein lysate was separated in precast SDS–polyacrylamide gels (Biorad 432,156) by electrophoresis and transferred onto PVDF membranes, followed by blocking in 5% w/v nonfat dry milk and incubation with the indicated antibodies (Additional file 1: Table S1) overnight. Proteins were detected with IRDye-conjugated secondary antibodies using the LI-COR Odyssey Imaging system and Image Studio v5.2.

### *RNAScope *in situ* hybridization*

RNA in situ hybridization was performed using the Multiplex Fluorescent V2 Assay kit (ACDBio) in combination with Opal Dyes (Akoya Biosciences) as per manufacturer’s instructions. The in situ probes are listed in the (Additional file 1: Table S3). Images of the optic nerve chiasm were acquired on an ICC50W fluorescent microscope with Leica Application Suite X software or with a Leica DMi8 fluorescent microscope using LAS X software.

### RNA sequencing and analysis

RNA from sham control, optic nerve crush, and TBI mice was isolated and sequenced on an Illumina HiSeq platform. RNA-seq reads were then aligned and quantitated to the Ensembl release 101 primary assembly with an Illumina DRAGEN Bio-IT on-premise server running version 3.9.3-8 software. Analyses were conducted using Partek Flow version 10.0. Bulk RNA-seq reads were qualified to annotation model mm10—Ensambl release 102 v2. All gene counts were normalized by median normalization. Differential genetic analysis was performed for differences between crush and control samples using DESeq2, excluding features with average coverage < 1. Differential genetic analysis results were filtered to include only genes with *p*-value ≤ 0.05 and fold change ≥ 2. Pathway analysis was then conducted using DAVID 2021, Knowledgebase v2022q4 with annotation pathways GOTERM_BP_DIRECT, GOTERM_CC_DIRECT, and GOTERM_MF_DIRECT. Excel was used to create a bar plot of the number of genes in selected pathways.

### In vivo* mouse treatments*

Four-week-old *Nf1*^OPG^ mice were treated with 10 mg/kg NFκB inhibitor (NFκB-IN; Caffeic acid phenethyl ester, Fisher Scientific, 274310), 275 mg/kg PLX3397-containing or control chow pellets (Free Base), 1 mg/ml anti-IL-1β neutralizing antibodies (R&D System 1060-DE-100), anti-IgG2a control antibodies (R&D Systems), or memantine hydrochloride (20 mg/kg) by intraperitoneal injection. Optic nerves were harvested when the mice reached 12 weeks of age, and the percentage of Ki67^+^ cells and volume measurements determined as previously reported [16]. At least 5 mice per group were used in all experiments.

### Quantification and statistical analysis

GraphPad Prism software was used for data analysis. To determine differences between two groups, a two-tailed Student’s *t* test was used, whereas multiple comparisons were analyzed by a one-way analysis of variance (ANOVA) test with Dunnett’s multiple comparisons. Statistical significance was set at *P* ≤ 0.05. All experiments were independently repeated at least three times with at least three biological replicates.

## Results

### Optic nerve crush induces optic glioma formation

**Fig. 1:**
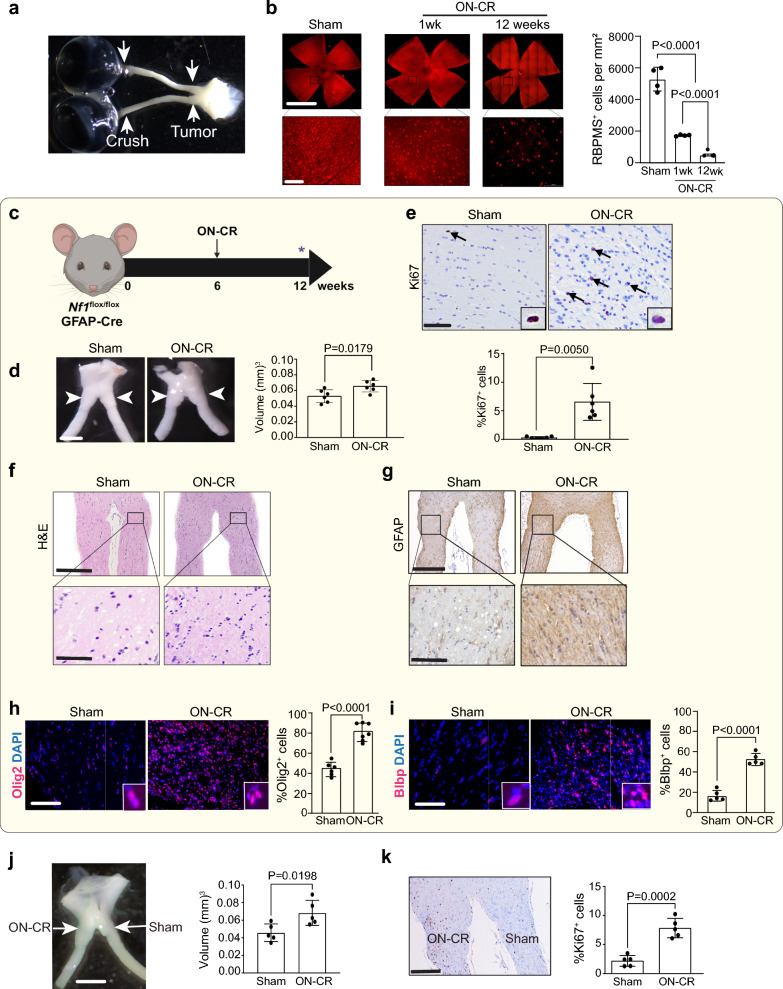
**a** Representative image of the site of optic nerve injury (ON-CR) relative to the site of tumor (optic glioma) formation. **b** Following optic nerve crush (ON-CR) at 6 weeks of age, *Nf1*^*flox/flox*^; hGFAP-Cre mice have progressive loss of retinal ganglion cells (RBPMS^+^ cells) in the retina relative to those undergoing a sham operation (sham, *n* = 4; 1 wk, *n* = 4; 12 wk, *n* = 3). **c** *Nf1*^*flox/flox*^; hGFAP-Cre mice are subjected to bilateral ON-CR, while control mice undergo a sham operation (incision without crush), at 6 weeks of age. Optic nerves are analyzed at 12 weeks of age. *Nf1*^*flox/flox*^; hGFAP-Cre mice following ON-CR have increased **d** optic nerve volumes (*n* = 6) and **e** percentages of proliferating (%Ki67^+^) tumor cells relative to the sham operation group (*n* = 6). Immunohistochemistry shows an increase in **f** optic nerve cellularity (H&E positive cells) and **g** GFAP expression in *Nf1*^*flox/flox*^; hGFAP-Cre mice following ON-CR compared to sham controls. Immunofluorescence microscopy reveals increased **h** %Olig2^+^ cells (sham, *n* = 6; ON-CR, *n* = 7) and **i** %Blbp^+^ cells (sham, *n* = 5; ON-CR, *n* = 5) in the optic nerves of *Nf1*^*flox/flox*^; hGFAP-Cre mice following ON-CR compared with sham controls. **j** Following unilateral optic nerve crush (u-ON-CR) in *Nf1*^*flox/flox*^; hGFAP-Cre mice at 6 weeks of age, the prechiasmatic region ipsilateral to the u-ON-CR exhibits greater **j** volume (*n* = 5) and **k** proliferation (%Ki67^+^ cells; *n* = 5) relative to the prechiasmatic region contralateral to the u-ON-CR at 12 weeks of age. Data are presented as the means ± SEM. Scale bars: **b** Upper panel scale bar, 500 µm; lower panel scale bar, 100 µm; **d**, **j** 100 µm; **f**, **g** Upper panel bar, 200 µm; lower panel scale bar, 50 µm; **e**, **h**, **i** 40 µm; **k** 200 µm, **b**, One-way ANOVA with Bonferroni post hoc correction; **d**, **e**, **h**, **i**, **j**, **k**, Two-tailed Student’s test

To determine whether optic nerve injury is sufficient to precipitate optic glioma formation in mice lacking *Nf1* expression in the cells of origin for these tumors (*Nf1*^*flox/flox*^; hGFAP-Cre mice), we performed bilateral optic nerve crush (ON-CR) at 6 weeks of age (Fig. 1a). Following ON-CR, there is progressive loss of retinal ganglion cells in the retina, as evidenced by reduced RPBMS^+^ cell (retinal ganglion cell; RGC) content (Fig. 1b). At 12 weeks of age, the optic nerves of *Nf1*^*flox/flox*^; hGFAP-Cre mice following ON-CR at 6 weeks of age exhibit increased optic nerve volume and proliferation (%Ki67^+^ cells) relative to sham surgery controls (Fig. 1c–e). Consistent with optic glioma formation, ON-CR results in increased cellularity (Fig. 1f) and GFAP (Glial fibrillary acidic protein; Fig. 1f, g) expression in the optic nerves of *Nf1*^*flox/flox*^; hGFAP-Cre mice, as well as increased Olig2^+^ (oligodendrocyte transcription factor 2) and Blbp^+^ (fatty acid binding protein 7) cellular content (%Olig2^+^ and %Blbp^+^ cells; Fig. 1h, i), relative to sham controls. It should be noted that tumors form in the prechiasmatic optic nerve and chiasm, which is 2.5 mm distal to the site of injury. Similarly, unilateral optic nerve crush (u-ON-CR) of *Nf1*^*flox/flox*^; hGFAP-Cre mice also results in optic glioma formation in the injured, but not in the contralateral, optic nerve (Fig. 1j, k**;** Fig S1a, b). In all cases, tumors are defined using human histologic criteria [4, 29] as a mass occupying lesion (architectural distortion, increased optic nerve volume) with increased proliferation (%Ki67^+^ cells), cellularity, and GFAP expression. The lesions generated in *Nf1*^*flox/flox*^; hGFAP-Cre mice following ON-CR are histologically indistinguishable from tumors that spontaneously develop in *Nf1*-OPG (*Nf1*^*flox/mut*^; hGFAP-Cre) mice. Importantly, ON-CR at 6 weeks of age results in optic glioma persistence at 24 weeks of age, as evidenced by increased optic nerve volume and proliferation (Fig S1c–e). In contrast, there is no change in optic nerve volume or proliferation in wild type mice following ON-CR performed at 6 weeks of age when analyzed at 12 weeks of age (Fig. S1f–h), indicating the requirement for *Nf1*-null preneoplastic cells to induce gliomagenesis in this injured, permissive microenvironment.

**Fig. 2:**
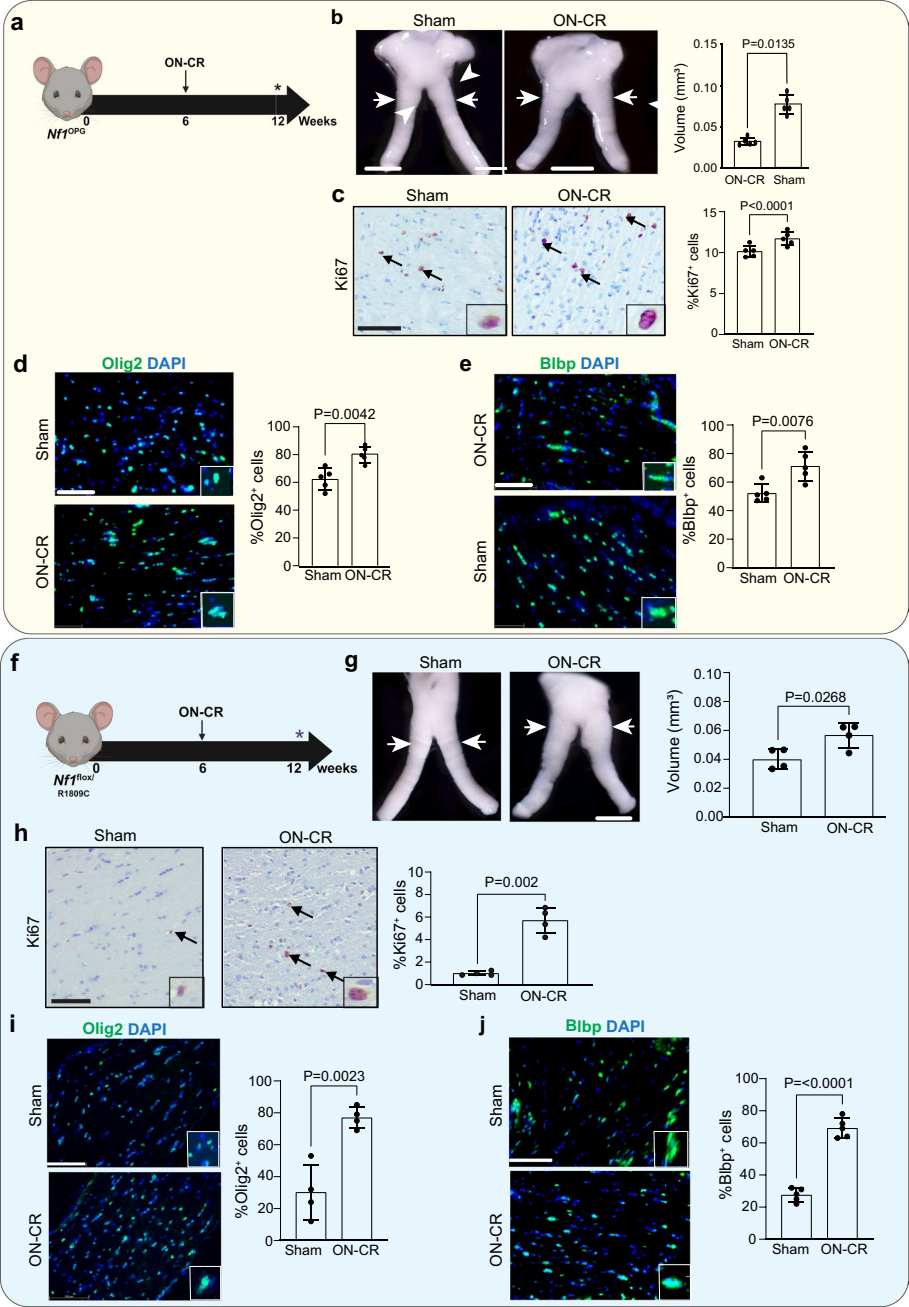
**a** *Nf1*^OPG^ mice undergo optic nerve crush (ON-CR) at 6 weeks of age, while optic nerves are analyzed at 12 weeks of age. *Nf1*^OPG^ mice following ON-CR have increased **b** optic nerve volumes and exhibit increased **c** proliferation (%Ki67^+^ cells; *n* = 5), **d** %Olig2^+^ cells (*n* = 7) and **e** %Blbp^+^ cells (*n* = 5). **f** 12-week-old *Nf1*^f/R1809C^; hGFAP-Cre mice following optic nerve crush (ON-CR) at 6 weeks of age exhibit increased **g** optic nerve volumes (*n* = 5), **h** proliferation (%Ki67^+^ cells, *n* = 5), **i** %Olig2^+^ cells (*n* = 7) and **j** %Blbp^+^ cells (*n* = 5) compared to those undergoing a sham operation (*n* = 4). Data are presented as the means ± SEM. Scale bars: **b**, **g** 100 µm; **c**, **d**, **e**, **h**, **i**, **j** 50 µm. Two-tailed Student’s *t* test

Next, we asked whether optic nerve injury worsens optic glioma growth in mice that normally form optic gliomas at 12 weeks of age (*Nf1*^flox/neo^; hGFAP-Cre, *Nf1*^OPG^ mice) [7]. *Nf1*^OPG^ mice harbor a germline *Nf1* gene mutation (neomycin targeted disruption of exon 31) and Cre-mediated *Nf1* loss in neuroglial progenitor cells at E16.5, which results in a well-defined temporal and spatial pattern of gliomagenesis [19, 55]. Following ON-CR at 6 weeks of age (Fig. 2a), *Nf1*^OPG^ mice exhibit increased optic nerve volumes (Fig. 2b), tumor proliferation (%Ki67^+^ cells; Fig. 2c), cell density (Additional file 2: Fig. S2a) and GFAP expression (Additional file 2: Fig. S2b) at 12 weeks of age, relative to sham controls. Moreover, the optic nerves of *Nf1*^OPG^ mice following ON-CR have increased Olig2^+^ and Blbp^+^ cell content (%Olig2^+^ and %Blbp^+^ cells; Fig. 2d, e, respectively) compared to the sham control group.

Lastly, we sought to determine whether optic gliomas could be induced in *Nf1*-mutant mice that normally do not form tumors. For this experiment, we leveraged mice harboring the germline Arg1809Cys (R1809C) *Nf1* missense mutation seen in people with NF1 who lack neurofibromas and brain tumors [50]. Similar to their human counterparts, *Nf1*^flox/R1809C^; hGFAP-Cre mice also do not form optic gliomas [5]. Following ON-CR at 6 weeks of age (Fig. 2f), *Nf1*^flox/R1809C^; hGFAP-Cre mice develop optic gliomas at 12 weeks of age with increased optic nerve volume (Fig. 2g), proliferation (Fig. 2h), Olig2^+^ and BLBP^+^ cellular content (%Olig2^+^ and %BLBP^+^ cells; Fig. 2i, j, respectively), as well as increased cellularity (Additional file 2: Fig. S2c) and GFAP expression (Additional file 2: Fig. S2d) compared to the sham control group. Taken together, these findings demonstrate that optic nerve crush is sufficient to induce glioma formation in mice with progenitor cell *Nf1* loss.

### T cells are not required for ON-CR-induced gliomagenesis

**Fig. 3:**
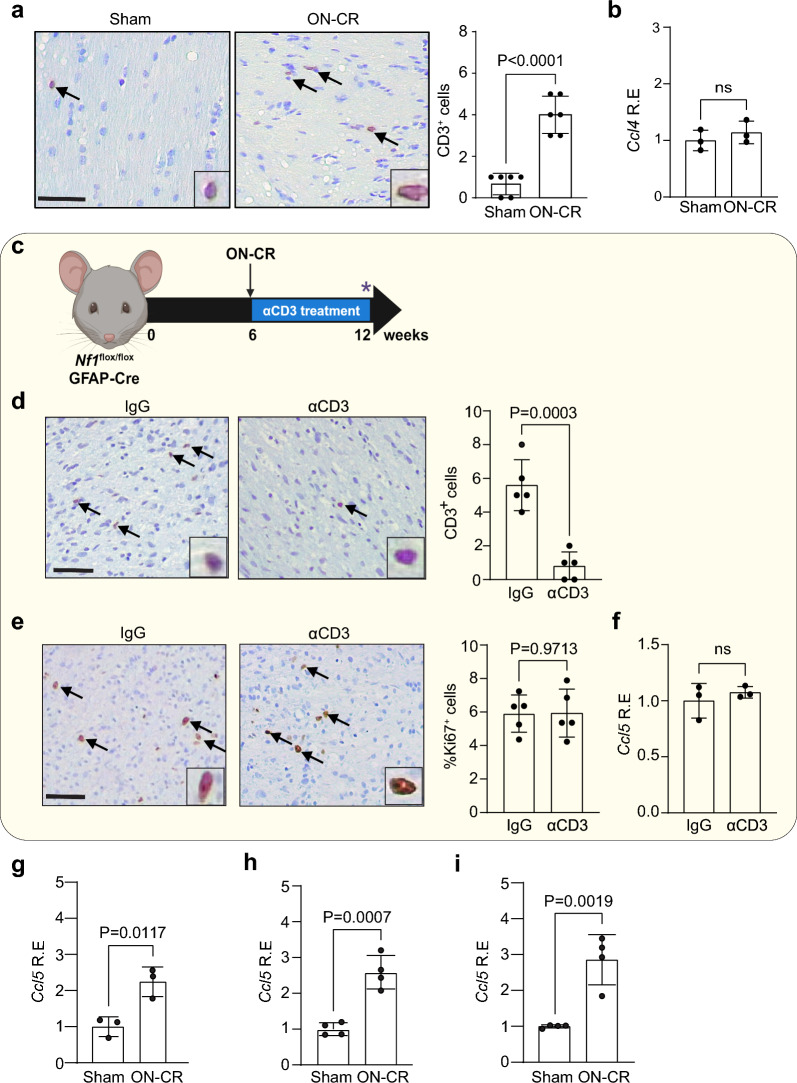
**a** Following optic nerve crush (ON-CR) at 6 weeks of age, *Nf1*^*flox/flox*^; hGFAP-Cre mice at 12 weeks of age have increased numbers of CD3^+^ cells in their optic nerves relative to those receiving a sham operation (*n* = 6); however, there was no change in **b** *Ccl4* mRNA expression by qPCR (*n* = 3). **c** Immediately after ON-CR, *Nf1*^*flox/flox*^; hGFAP-Cre mice received intraperitoneal injections of anti-CD3 (αCD3) antibodies every other day for 6 weeks. Control mice were injected with anti-IgG antibodies. Optic nerves were analyzed at 12 weeks of age. αCD3 antibody treatment (150 µg) reduced **d** CD3^+^ cell content (*n* = 5) and **e** proliferation (%Ki67^+^ cells; *n* = 5). **f** αCD3 antibody treatment following ON-CR does not change *Ccl5* RNA expression by qPCR compared to IgG-treated controls (150 µg; *n* = 3). Increased *Ccl5* RNA expression in the optic nerves (qPCR) was observed 7 days after ON-CR in **g** *Nf1*^*flox/flox*^; hGFAP-Cre (*n* = 3), **h** athymic (*Foxn1*^nu/nu^; *n* = 4), and **i** *Rag1*^−/−^ mice (*n* = 4) compared to sham controls. Scale bars: **a**, **d**, **e** 50 µm. Two-tailed Student’s *t* test (ns, not significant)

Previous studies have implicated T cells in the pathobiology of optic nerve injury [12, 18, 59, 63]. Similarly, we have previously demonstrated that T cells are required for optic glioma formation and growth in experimental *Nf1* mouse models [16, 19, 28, 43]. In *Nf1*^OPG^ mice, we elucidated a “neuron-immune-cancer cell” circuit [28], where T cells are induced by *Nf1*-mutant neuron-produced midkine to express Ccl4, which stimulates TAMs to secrete Ccl5, a key growth factor for *Nf1*-OPG development and progression [28, 53]. To determine whether T cells are necessary for *Nf1* optic gliomagenesis in *Nf1*^*flox/flox*^; hGFAP-Cre mice in the setting of ON-CR, we performed several experiments. First, we analyzed T cell content in the optic nerves of *Nf1*^*flox/flox*^; hGFAP-Cre mice following ON-CR. While increased CD3^+^ cell content is observed (Fig. 3a), there is no increase in *Ccl4* expression as measured by qPCR (Fig. 3b). Second, we depleted T cells with systemic anti-CD3 (αCD3) antibody treatment for 6 weeks immediately after ON-CR (6 weeks of age) and compared to mice treated with anti-IgG control antibodies (Fig. 3c). In contrast to our prior studies in which T cell depletion blocked *Nf1*-OPG growth and reduced CD3 T cell content [28], CD3 T cell depletion (Fig. 3d) does not change optic nerve proliferation (Fig. 3e) or *Ccl5* expression (Fig. 3f) in 12-week-old *Nf1*^*flox/flox*^; hGFAP-Cre mice following ON-CR at 6 weeks of age relative to controls. Third, since T cells stimulate TAM production of Ccl5 to sustain *Nf1*-OPG growth [28, 43], we quantified *Ccl5* induction following ON-CR at 6 weeks of age. ON-CR in wild-type mice leads to increased Ccl5 production 7 days post-injury (Fig. 3g). However, this induction does not require T cells, as optic nerves from mice lacking mature T cells (*Foxn1*^*−/−*^; Fig. 3h) or all mature adaptive immune cells (*Rag1*^*−/−*^; Fig. 3i) still exhibit increased Ccl5 expression 7 days after injury. In contrast to the requirement for T cells for TAM Ccl5 production during spontaneous gliomagenesis in *Nf1*^OPG^ mice, optic nerve injury-induced Ccl5 expression operates in a T cell-independent manner.

### TAM Ccl5 production is required for ON-CR-induced gliomagenesis

**Fig. 4:**
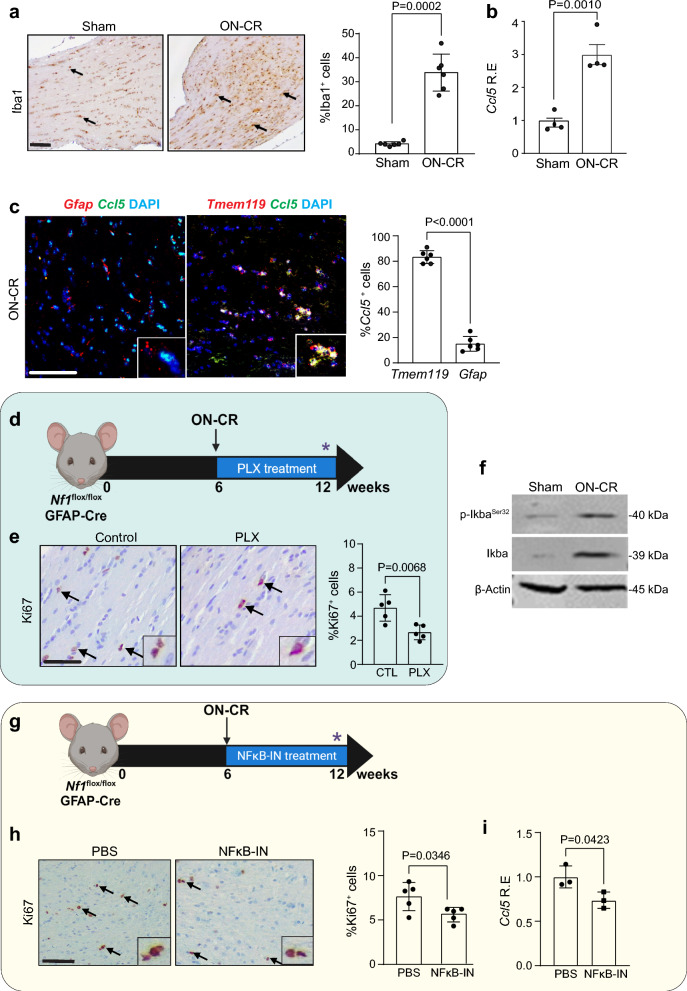
Following optic nerve crush (ON-CR) at 6 weeks of age, *Nf1*^*flox/flox*^; hGFAP-Cre mice at 12 weeks of age have increased **a** TAMs (%Iba1^+^ cells; *n* = 6) and **b** *Ccl5* mRNA expression (qPCR; *n* = 4) in their optic nerves relative to sham controls. **c** *Ccl5* is expressed by Tmem119^+^ microglia, but not by astrocytes (GFAP^+^ cells). **d** Immediately after ON-CR at 6 weeks of age, *Nf1*^*flox/flox*^; hGFAP-Cre mice received PLX33397 (PLX) by replacing normal chow (AIN-76A rodent diet; Research Diet Inc.) with 275 mg/kg PLX3397-containing chow (Free Base) for 6 weeks. Control mice received normal chow. Optic nerves were analyzed at 12 weeks of age. **e** PLX treatment results in reduced optic nerve proliferation (%Ki67^+^ cells; *n* = 5). **f** Following ON-CR, *Nf1*^*flox/flox*^; hGFAP-Cre mouse optic nerves (*n* = 3) have increased Iκbα phosphorylation relative to sham controls (*n* = 3). **g** Treatment of *Nf1*^*flox/flox*^; hGFAP-Cre mice with 10 mg/kg CAPE (NFκB-IN) immediately after ON-CR at 6 weeks of age results in reduced **h** optic nerve proliferation (Ki67^+^ cells; *n* = 5) and **i** *Ccl5* RNA expression (*n* = 3) relative to vehicle-treated mice at 12 weeks of age. Data are presented as the means ± SEM. Scale bars: **a**, **c**, **e**, **h**. 50 µm. Two-tailed Student’s *t* test

Consistent with our prior reports demonstrating that TAMs and TAM-produced Ccl5 are required for *Nf1* optic glioma formation and growth [28, 46, 53], *Nf1*^*flox/flox*^; hGFAP-Cre mouse ON-CR results in a dramatic increase in TAM content (%Iba1^+^ cells; Fig. 4a) and Ccl5 expression (Fig. 4b) relative to sham treated mice. The majority of the Ccl5 is made by TAMs (Additional file 2: Fig. S3a, b), rather than astrocytes, as revealed by *Tmem119* and *Ccl5* RNAScope (Fig. 4c). To demonstrate that TAMs are required for ON-CR-induced optic glioma formation in *Nf1*^*flox/flox*^; hGFAP-Cre mice, we performed two experiments. First, we depleted TAMs using the CSF-1R inhibitor PLX3397 (PLX) for 6 weeks immediately following ON-CR at 6 weeks of age (Fig. 4d). TAM depletion results in reduced tumor proliferation (%Ki67^+^ cells; Fig. 4e), Blbp^+^ and Olig2^+^ cell content (%Olig2^+^ and %Blbp^+^ cells; Additional file 2: Fig. S3c) and Ccl5 expression (Additional file 2: Fig. S3d). Since TAM Ccl5 production is regulated by NFκB activation in the setting of *Nf1*-OPG [16, 28], we analyzed Iκbα phosphorylation (p-Iκba^Ser32^) by western blotting. At 12 weeks of age, there is increased Iκbα phosphorylation in the optic nerves of *Nf1*^OPG^ mice following ON-CR relative to sham controls (Fig. 4f), which is restricted to TAMs (Additional file 2: Fig. S3e). Second, since ON-CR induces TAM Ccl5 production, we treated *Nf1*^*flox/flox*^; hGFAP-Cre mice with an NFκB inhibitor (10 mg/kg CAPE; NFκB-IN) for six weeks following ON-CR at 6 weeks of age (Fig. 4g). Relative to vehicle (PBS)-treated controls, NFκB inhibition reduces tumor proliferation (%Ki67^+^ cells; Fig. 4h), BLBP^+^ and Olig2^+^ cells (%Olig2^+^ and %Blbp^+^ cells; Additional file 2: Fig. S3f) and Ccl5 expression (Fig. 4i) at 12 weeks of age. Collectively, these findings demonstrate that ON-CR induces *Nf1* optic gliomagenesis in a TAM/Ccl5-dependent manner.

### Injury-induced IL-1β increases TAM production of Ccl5

**Fig. 5:**
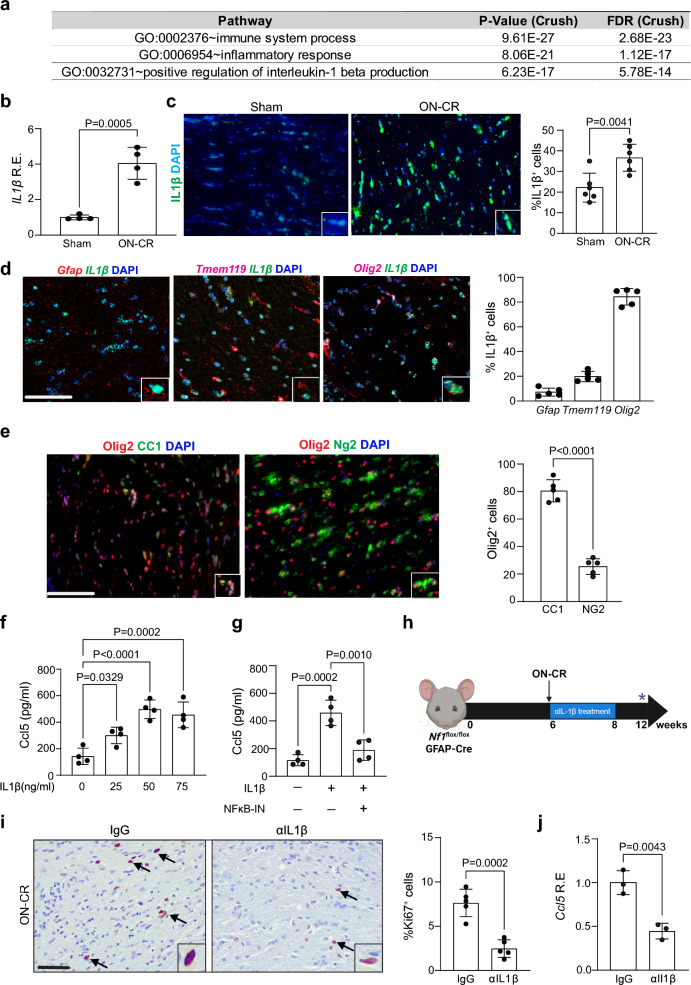
**a** Top 3 identified pathways enriched in the optic nerves of *Nf1*^*flox/flox*^; hGFAP-Cre mice after optic nerve crush (ON-CR) relative to sham controls following filtering of differentially expressed transcripts from bulk RNA sequencing. **b** Increased *IL-1β* RNA expression (qPCR) is observed in the optic nerves of 12-week-old *Nf1*^*flox/flox*^; hGFAP-Cre mice following ON-CR at 6 weeks of age relative to sham controls (*n* = 4). **c** Representative IL-1β immunostaining in the optic nerves of *Nf1*^*flox/flox*^; hGFAP-Cre mice following ON-CR relative to sham control mice. **d** RNAscope (in situ RNA hybridization) demonstrates that oligodendrocytes (Olig2^+^ cells) express IL-1β. **e** Immunostaining reveals that the majority of the Olig2^+^ cells co-label with CC1, but not with NG2. **f** Increasing IL-1β concentrations (0–75 ng/ml) increases microglia Ccl5 protein expression in vitro. **g** IL-1β-induced microglial Ccl5 production is attenuated by 10 mg/kg NFκB inhibitor (NFκB-IN; Caffeic acid phenethyl ester) treatment in vitro (*n* = 4). **h** Anti-IL1β neutralizing antibody treatment (1 mg/ml) of *Nf1*^*flox/flox*^; hGFAP-Cre mice immediately after ON-CR at 6 weeks of age results in decreased **i** optic nerve proliferation (%Ki67^+^ cells; *n* = 5) and **j** *Ccl5* mRNA expression (qPCR; *n* = 3) when analyzed at 12 weeks of age compared to IgG-treated (1 mg/ml) controls. Data are presented as the means ± SEM. Scale bars: **c**, **d**, **e**, **i** 50 µm. **b**, **c**, **e**, **i**, **j**. Two-tailed Student’s *t* test; **f**, **g** One-way ANOVA with Bonferroni post-test correction

To investigate the mechanism by which ON-CR induces TAM Ccl5 production, we performed bulk RNA sequencing on isolated optic nerves from ON-CR and sham-treated *Nf1*^*flox/flox*^; hGFAP-Cre mice (Additional file 2: Fig. S4a). Following filtering of differentially expressed transcripts (*P* values ≤ 0.01, false discovery rate ≤ 0.05, and log fold change ≥ 5), we examined the top 40 differentially regulated pathways (Additional file 2: Fig. S4b). Based on a previous report demonstrating a critical role for IL-1β in traumatic brain injury [30] and “positive regulation of IL-1β production” pathway emerging as one of the top pathways identified in our sequencing analysis (Fig. 5a), we examined IL-1β expression following ON-CR in *Nf1*^*flox/flox*^; hGFAP-Cre mice. Increased RNA (Fig. 5b) and protein (Fig. 5c) expression is observed in the optic nerves of *Nf1*^*flox/flox*^; hGFAP-Cre mice following ON-CR relative to sham controls. RNAscope reveals that 80% of the IL-1β^+^ cells were oligodendrocytes (Olig2^+^, CC1^+^ cells), rather than astrocytes or TAMs (Fig. 5d, e).

To demonstrate that IL-1β is *sufficient* to increase TAM Ccl5 production, we showed that IL-1β exposure increases microglia production of Ccl5 protein in a dose-dependent manner in vitro (Fig. 5f). This induction by IL-1β in microglia is abrogated following treatment with an NFκB inhibitor (CAPE; 100 µM, NFκB-IN; Fig. 5g). To determine whether IL-1β is *necessary* for ON-CR-induced optic gliomagenesis, *Nf1*^*flox/flox*^; hGFAP-Cre mice were administered anti-IL1β (αIL-1β) neutralizing antibodies for two weeks immediately following ON-CR at 6 weeks of age (Fig. 5h). Consistent with an obligatory role for IL-1β in injury-induced optic glioma formation, intraperitoneal αIL1β antibody treatment reduces optic nerve cellular proliferation (Fig. 5i), Blbp^+^ and Olig2^+^ cell content (%Olig2^+^ and %Blbp^+^ cells; Additional file 2: Fig. S4c, d, respectively) and Ccl5 expression (Fig. 5j) at 12 weeks of age relative to IgG control-treated mice.

### Neuronal glutamate increases oligodendrocyte IL-1β production

**Fig. 6:**
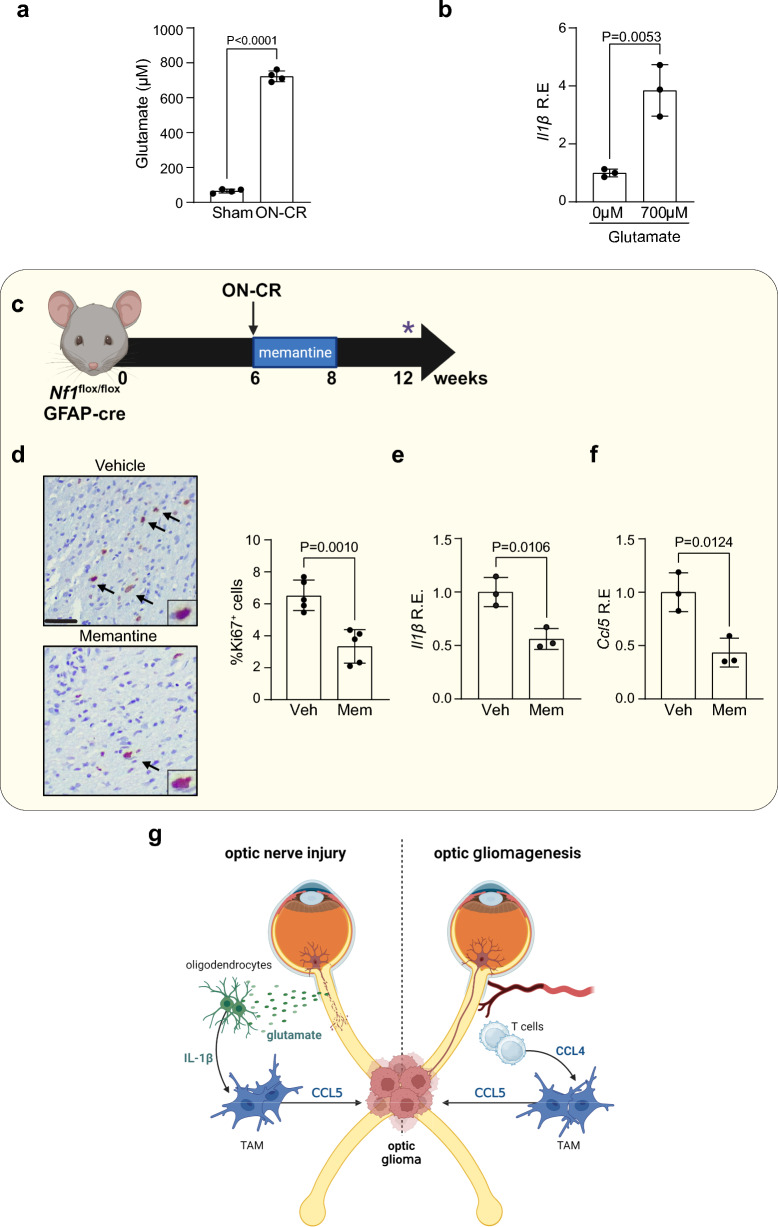
Increased **a** glutamate (ELISA) and **b** *Il-1β* mRNA (qPCR) expression in the optic nerves of *Nf1*^*flox/flox*^; hGFAP-Cre mice at 3 months of age following optic nerve crush at 6 weeks of age (ON-CR). **c** Memantine hydrochloride treatment (20 mg/kg) of *Nf1*^*flox/flox*^; hGFAP-Cre mice for two weeks immediately after ON-CR at 6 weeks of age reduces **d** optic nerve proliferation (%Ki67^+^ cells; *n* = 5) when analysed at 12 weeks of age. Memantine treatment decreases **e** *Il-1β* and **f** *Ccl5* mRNA expression (qPCR; *n* = 3). **g** Mechanistic model comparing the cellular and molecular events that induce gliomagenesis following injury (left side) and during spontaneous tumor formation (right side). (LEFT) ON-CR induces retinal ganglion cell glutamate release, which stimulates oligodendrocytes to release IL-1β, resulting in NFκB-dependent TAM Ccl5 expression and culminating in *Nf1*-OPG formation and growth. (RIGHT) T cells are induced to express Ccl4, which stimulates TAM production of Ccl5 and *Nf1*-OPG formation and growth. Data are presented as the means ± SEM. Scale bars: **d** 50 µm. Two-tailed Student’s *t* test

To define the mechanism underlying oligodendrocyte IL-1β production, we asked whether neuronal glutamate could be the etiologic cause. Elevated glutamate levels have been reported in several studies of experimental brain and optic nerve injury in mice [6, 26, 57, 62]. Consistent with these prior reports, glutamate levels are elevated (~ 700 µM) in the optic nerves of ON-CR, compared to sham control, *Nf1*^*flox/flox*^; hGFAP-Cre mice (Fig. 6a). Using this same concentration, glutamate induces oligodendrocyte *Il1β* RNA expression in vitro (Fig. 6b). Moreover, blocking glutamate receptor function with memantine in *Nf1*^*flox/flox*^; hGFAP-Cre mice following ON-CR at 6 weeks of age (Fig. 6c) results in reduced tumor proliferation (%Ki67^+^ cells; Fig. 6d), as well as reduced expression of optic nerve *Il1β* (Fig. 6e) and *Ccl5* (Fig. 6f). Taken together, these results establish a paracrine circuit in which optic nerve neuronal injury results in glutamate release, oligodendrocyte IL-1β and TAM Ccl5 production, and tumor formation (Fig. 6g).

### Traumatic brain injury induces optic glioma through the same paracrine circuit

**Fig. 7:**
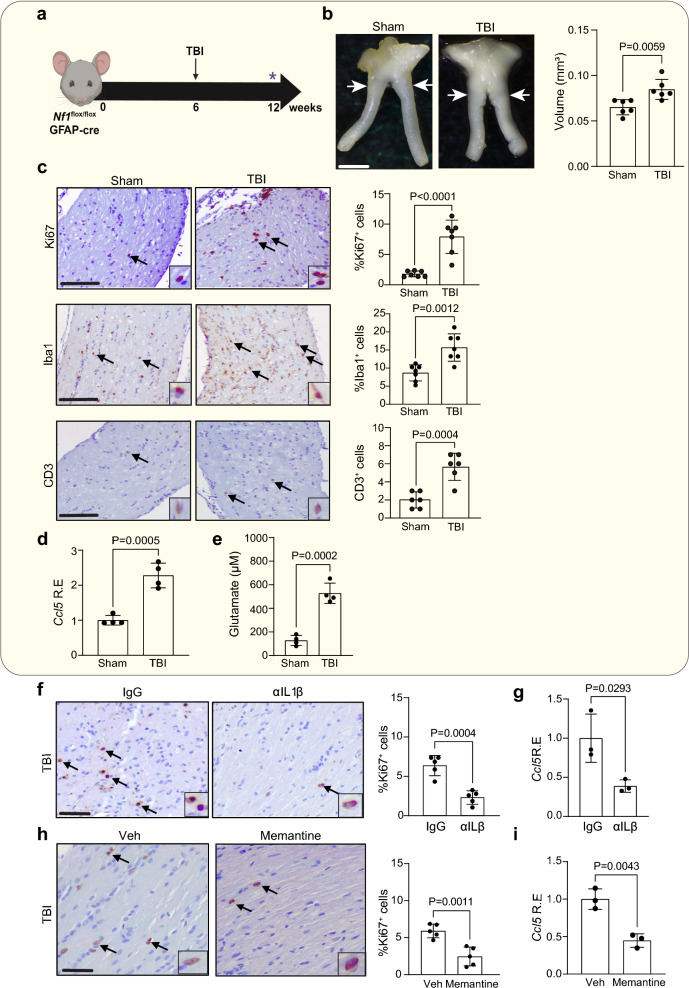
**a** Traumatic brain injury (TBI) in *Nf1*^*flox/flox*^; hGFAP-Cre mice at 6 weeks of age results in increased **b** optic nerve volume (*n* = 6) and **c** proliferation (%Ki67^+^ cells; *n* = 7) relative to sham treated mice when analyzed at 12 weeks of age, as well as increased TAMs (%Iba1^+^ cells) and CD3^+^ T cell content. Increased **d** *Ccl5* RNA expression (qPCR) and **e** glutamate levels are observed in the optic nerves of *Nf1*^*flox/flox*^; hGFAP-Cre mice (*n* = 4) 7 days after TBI compared to sham controls. **f** αIL-1β neutralizing antibody (1 mg/ml) and **h** memantine treatment (20 mg/kg) both decrease proliferation (%Ki67^+^ cells; *n* = 5) and **g**, **i** Ccl5 expression (*n* = 3) following TBI relative to their respective control mice (IgG and vehicle treatment groups). Data are presented as the means ± SEM. Scale bar: **b** 100 μm; **c**, **f**, **i** 50 μm. Two-tailed Student’s *t* test

To extend these findings to another model of optic nerve injury, we sought to determine whether diffuse closed-head traumatic brain injury (TBI)-mediated optic nerve injury would also result in optic glioma formation. The experimental TBI model (modCHIMERA) also results in multiregional, prolonged neuroinflammation [51], which lasts into the chronic post-injury period (> 1mo post-injury). Following TBI, there is clear damage to the optic nerve, as evidenced by increased β-APP and SMI-32 expression in the optic nerves 7 days post injury (Additional file 2: Fig. S5a) [31]. TBI was therefore performed on *Nf1*^*flox/flox*^; hGFAP-Cre mice at 6 weeks of age for analysis at 12 weeks of age (Fig. 7a). Similar to ON-CR, *Nf1*^*flox/flox*^; hGFAP-Cre mice have increased optic nerve volumes (Fig. 7b) and proliferation (%Ki67^+^ cells; Fig. 7c) following TBI compared to sham-treated controls, with increased optic nerve cellularity (Additional file 2: Fig. S5b), GFAP immunoreactivity (Additional file 2: Fig. S5c), and Olig2^+^ and Blbp^+^ cell content (%Olig2^+^ and %Blbp^+^ cells; Additional file 2: Fig. S5d, e, respectively). In addition, TBI results in increased TAMs (%Iba1^+^ cells) and T lymphocytes (CD3^+^ cells) (Fig. 7c), elevated *Ccl5* expression (Fig. 7d) and higher glutamate levels (Fig. 7e). As observed following ON-CR, optic gliomagenesis after TBI in *Nf1*^*flox/flox*^; hGFAP-Cre mice is blocked by treatment with αIL-1β antibodies or memantine. In this regard, there is reduced tumor proliferation (%Ki67^+^ cells; Fig. 7f) and *Ccl5* expression (Fig. 7fg) following systemic αIL-1β antibody treatment relative to IgG antibody treatment, whereas *Nf1*^*flox/flox*^; hGFAP-Cre mice given memantine following TBI had decreased tumor proliferation (%Ki67^+^ cells; Fig. 7h) and *Ccl5* expression (Fig. 7i) relative to vehicle-treated control mice. Collectively, these experiments establish a new paracrine factor cellular circuit (“neuron-glia” circuit) generated by CNS injury that converges on TAMs to induce *Nf1* optic gliomagenesis.

## Discussion

The concept that nervous system injury and nervous system tumor formation each induce immune (“inflammatory”) responses in the local tissues suggests the existence of overlapping mechanisms underlying the pathobiology of brain damage and tumorigenesis. In this regard, prior work focused on peripheral nervous system tumors in NF1 (neurofibromas) have shown that neurofibromas develop specifically at wound sites in mice with embryonic *Nf1* loss in Schwann cell progenitors [49]. These injury-induced tumors likewise exhibit increased T cell and monocyte infiltration; however, the mechanism responsible for tumorigenesis was not fully elucidated. Leveraging several *Nf1* GEM strains and two complementary optic nerve injury models, we now demonstrate that optic nerve damage is sufficient to induce the formation of gliomas. We further define a glutamate-dependent neuron-oligodendrocyte interaction that results in IL-1β-mediated TAM production of Ccl5 production and tumor formation. These observations raise several key points.

First, optic nerve damage and TBI both result in the release of glutamate that acts on established physiologic interactions between neurons and oligodendrocytes in the normal brain. Receptors for α-amino-3-hydroxy-5-methyl-4-isoxazolepropionic acid (AMPA) are expressed by both neurons and glia in the central and peripheral nervous system [35], where loss of AMPA receptor function leads to increased axon and myelin damage in the setting of demyelinating disease [20]. Additionally, neuron to oligodendrocyte precursor synapses are critical for proper oligodendrocyte development and myelination [9, 36, 40]. Moreover, in the setting of brain tumors, glutamatergic synaptic input to glioma cells drives malignant glioma progression [56], whereas increased neuronal excitability resulting from light exposure leads to the secretion of ADAM10 that cleaves membrane-bound neuroligin-3 on oligodendrocyte precursors to induce *Nf1* optic glioma initiation and growth [42]. The repurposing of normal brain cellular interactions to induce cancer supports the idea that tumorigenesis usurps some of the brain cellular circuitry important for normal nervous system development and maintenance.

Second, the finding that the immune circuits required for *Nf1*-OPG formation and progression can serve as convergence points for brain tumor risk factors provides a contextual framework for understanding cancer at a circuit level. In *Nf1*-OPG tumors, increased neuronal excitability as a consequence of *Nf1* mutation results in midkine production, which stimulates T cells to make Ccl4 and induce TAM Ccl5 production [5, 28]. This “neuron-immune-cancer” cell axis establishes a circuit whose interruption blocks tumor formation and progression [5, 19], but also could be modified by systemic changes that alter T cell function. As such, one risk modifier, asthma, reduces glioma incidence in children with NF1 [47] and in *Nf1*-OPG mice [16]. In an analogous manner, we now show that brain injury converges on TAM Ccl5 production through a different mechanism involving neuronal glutamate-induced oligodendrocyte IL-1β secretion, a key immunomodulator of brain injury [2, 21]. Based on these findings, we hypothesize that other risk factors, like the gut microbiota or other systemic exposures, might similarly modify tumorigenesis through convergence on these stromal circuits.

Third, while the relationship between brain injury and glioma progression in people is controversial [37], it is conceivable that changes in the local microenvironment, such as those induced in the setting of injury, could act on preneoplastic cells in otherwise healthy individuals to initiative tumorigenesis. In this regard, it is now appreciated that children and adults are genetic mosaics for numerous somatic mutations affecting genes involved in brain tumor development [41, 44, 58]. Specifically, double-strand breaks and error-prone repair create genomic deletions are found in neurotypical individuals across the lifespan [34], which develop in spatially distinct brain regions that reflect the timing of their acquisition during development [13]. Importantly, somatic single nucleotide variants, including the *IDH1*^R32H^ mutation, which typifies low-grade astrocytoma [45, 60], as well as pathogenic *NF1* variants, are detected in the non-diseased human brain [24]. Given the presence of these potentially susceptible preneoplastic cells harboring somatic cancer-associated genetic mutations, studies using experimental models of other cancer predisposition syndromes will be required to establish a mechanistic relationship between injury and tumorigenesis.

## Conclusion

Using two distinct brain injury paradigms and several different genetically engineered mouse strains, we establish that CNS injury is sufficient to induce glioma formation in mice harboring *Nf1*-deficient neuroglial progenitor cells. We further define the mechanistic etiology for injury-induced tumorigenesis, demonstrating that non-neoplastic stromal alterations secondary to injury create a microenvironment supportive of tumor development. These findings raise the intriguing possibility that local changes in cellular signaling resulting from CNS insults overlap with those stromal circuits required for brain tumor initiation and progression.

### Supplementary Information

**Additional file 1**. **Supplementary Tables. Table S1.** Antibodies used. **Table S2.** qRT-PCR probes used. **Table S3.** RNAScope probes used.

**Additional file 2**. **Supplementary Figures. Fig. S1.** Following unilateral optic nerve crush (u-ON-CR) performed at 6 weeks of age, the prechiasmatic region of *Nf1*^*flox/flox*^; hGFAP-Cre mice ipsilateral to the u-ON-CR exhibits (**a**) greater cellularity and (**b**) GFAP expression at 12 weeks of age. (**c**) Optic gliomas persist at 24 weeks of age in *Nf1*^*flox/flox*^; hGFAP-Cre mice following optic nerve crush performed at 6 weeks of age, as evidenced by increased (**d**) optic nerve volume and (**e**) proliferation (%Ki67^+^ cells) (*n*=5). (**f**) Following optic nerve crush (ON-CR) at 6 weeks of age, wild type (*Nf1*^flox/flox^) mice exhibit no change in (**g**) optic nerve volume (*n *= 5) or (**h**) proliferation (%Ki67^+^ cells; sham, *n* = 7; ON-CR, *n* = 6) when analyzed at 12 weeks of age. Scale bars: **a, b** Upper panel scale bar, 200 μm; lower panel scale bar, 50 μm **d, g** 100 μm;** e, h** 50µm. Two-tailed Student’s *t* test (ns, not significant). Asterisks denote the age at euthanasia and analysis. **Fig. S2.** Representative images of *Nf1*^OPG^ mouse optic nerves following ON-CR at 6 weeks of age have increased (**a**) cellularity and (**b**) GFAP expression relative to the sham operation group. Optic nerves from *Nf1*^f/R1809C^; hGFAP-Cre mice following optic nerve crush at 6 weeks of age have increased (**c**) cellularity and (**d**) GFAP expression compared to the sham operation group when analyzed at 12 weeks of age. Scale bars: **a-d **upper panel scale bar, 200 μm; lower panel scale bar, 50 μm. **Fig. S3.** Following ON-CR at 6 weeks of age, RNAscope reveals increased numbers of (**a**) *Tmem119*^*+*^ cells and (**b**)** Ccl5**^+^ cells in the optic nerves of *Nf1*^*flox/flox*^; hGFAP-Cre mice at 12 weeks of age. PLX3397 (275mg/kg PLX) treatment reduces ON-CR-induced increases (**c**) in Blbp^+^ and Olig2^+^ cell content (%Blbp^+^, %Olig2^+^ cells; *n*=5), as well as (**d**) *Ccl5* expression, in the optic nerves at 12 weeks of age relative to those fed the control (CTL) diet (*n*=3). (**e**) TAMs (Iba1^+^ cells) in 12-week-old *Nf1*^*flox/flox*^; hGFAP-Cre mice express p65-NFκB following ON-CR at 6 weeks of age. *Nf1*^*flox/flox*^; hGFAP-Cre mice that underwent ON-CR at 6 weeks of age exhibit reduced Blbp^+^ and Olig2^+^ (%Blbp^+^, %Olig2^+^ cells; *n*=5) cell content at 12 weeks of age following (**f**) Caffeic acid phenethyl (10mg/kg CAPE, NFκB inhibitor, NFκB-IN) treatment relative to PBS-treated controls. Data are presented as the means ± SEM. Scale bars: **a, b, e, f, g **50 μm; Two-tailed Student’s *t* test. **Fig. S4. **(**a**) Analysis of differential gene expression by bulk RNA sequencing of optic nerves from 12-week-old *Nf1*^*flox/flox*^; hGFAP-Cre mice following optic nerve crush (ON-CR; n=3) or sham surgery (n=3) at 6 weeks of age. (**b**) R studio version 2023.03.0 was used to identify enriched pathways and to calculate the mean and p-value for ON-CR relative to sham mouse groups, as well as to determine the FDR for each shared pathway. The 40 most significant pathways were identified using the mean FDR ≤ 0.05, and log fold changes greater or equal to ±5. (**c, d**) αIL1β treatment after ON-CR at 6 weeks of age results in reduced optic nerve Blbp^+^ and Olig2^+^ cell content in *Nf1*^*flox/flox*^; hGFAP-Cre mice at 12 weeks of age compared to IgG controls (%Blbp^+^, %Olig2^+^ cells; *n*=5). Data are presented as the means ± SEM. Scale bars: **c, d **50μm; Two-tailed Student’s *t* test. **Fig. S5. **(**a**) Increased β-APP and SMI-32 expression is seen in the optic nerves of *Nf1*^*flox/flox*^; hGFAP-Cre mice 7 days after TBI (modCHIMERA). Following TBI at 6 weeks of age, increased (**b**) optic nerve cellularity and (**c**) GFAP expression is observed by immunohistochemistry in *Nf1*^*flox/flox*^; hGFAP-Cre mice at 12 weeks of age relative to sham controls. Immunofluorescence reveals increased (**d**) %Blbp^+^ (*n* = 5) and (**e**) %Olig2^+^ (*n *= 5) cells in *Nf1*^*flox/flox*^; hGFAP-Cre mice at 3 months of age following TBI at 6 weeks of age compared to sham injury controls. Data are presented as the means ± SEM. Scale bars: **a** 50 μm,** b, c** upper panel bar, 200 μm; lower panel scale bar, 50 μm, **d, e** 40μm. Two-tailed Student’s *t* test.

## Data Availability

Bulk RNA sequencing data was deposited in GEO (accession #GSE233373). All other laboratory-generated resources will be made available upon request.

## Abbreviations

AMPA: α-Amino-3-hydroxy-5-methyl-4-isoxazolepropionic acid
Blbp: Fatty acid binding protein 7
CNS: Central nervous system
GEM: Genetically engineered mouse
GFAP: Glial fibrillary acidic protein
MYC: Myc proto-oncogene
NF1: Neurofibromatosis type 1
*Nf1*^OPG^: *Nf1*^flox/mut^; GFAP-Cre
NFκB-IN: NFκB inhibitor
OPG: Optic pathway glioma
ON-CR: Optic nerve crush
Olig2: Oligodendrocyte transcription factor 2
TAM: Tumor-associated monocytes
TBI: Traumatic brain injury
u-ON-CR: Unilateral optic nerve crush
