# Supplementary material for: Brain injury drives optic glioma formation through neuron-glia signaling
Source: Acta Neuropathol Commun. 2024 Feb 2;12:21. doi: 10.1186/s40478-024-01735-w (PMC10837936; doi:10.1186/s40478-024-01735-w)
Supplement: Supplementary file 1 — Additional file 1. Supplementary Tables. Table S1. Antibodies used. Table S2. qRT-PCR probes used. Table S3. RNAScope probes used. [file 40478_2024_1735_MOESM1_ESM.docx]

**Table S1. Antibodies used.**

| **Antibody** | **Host** | **Source** | **Dilution** |
| --- | --- | --- | --- |
| β-Actin (WB) | mouse | Cell Signaling, 58169S | 1:1000 |
| β-APP (IF) | rabbit | Thermo 36-6900 | 1:300 |
| BLBP (IF) | rabbit | EMD Millipore, 3439833 | 1:400 |
| CC1(IF) | Mouse | EMD Millipore, OP80 | 1:200 |
| CD3 (IHC) | rat | Abcam, 11089 | 1:50 |
| GFAP (IHC) | rat | Fisher scientific 13-0300 | 1:500 |
| Iba1 (IF) | mouse | EMD Millipore, MABN92 | 1:400 |
| Iba1 (IHC) | rabbit | Wako, 019-19741 | 1:500 |
| IκBα (WB) | rabbit | Cell Signaling, 4812S | 1:1000 |
| IL-1β (IHC, IF) | rabbit | Abcam, ab9722 | 1:200 |
| Ki67 (IHC) | mouse | BD Pharmingen, 550609 | 1:400 |
| NG2 (IF) | rat | R&D systems  (MAB6689-SP) | 1:200 |
| Olig2 (IF) | rabbit | Novus, NBP1-28667 | 1:500 |
| p65-NFκB (IF) | Rabbit | Abcam, ab32536 | 1:500 |
| Phospho-IκBα (WB) | rabbit | Cell Signaling, 2859S | 1:1000 |
| RBPMS (IF) | guinea pig | Phospho solutions 1832-RBPMS | 1:200 |
| SMI-32 (IF) | mouse | Biolegend 801701 | 1:1000 |

**Table S2. qRT-PCR probes used.**

| **Gene** | **Probe set** |
| --- | --- |
| *Ccl4*  (mouse) | Mm00443111_m1(TaqMan Gene Expression) |
| *Ccl5*  (mouse) | Mm01302427_m1(TaqMan Gene Expression) |
| *Gapdh*  (mouse) | Mm99999915_g1 (TaqMan Gene Expression); internal control |
| *Il1β*  (mouse) | Mm00434228_m1(TaqMan Gene Expression |

**Table S3. RNAScope probes used.**

| **Probe**  **(RNAscope)** | **Source** | **Dilution** |
| --- | --- | --- |
| *Ccl5* | Advanced Cell Diagnostics, 469601 | 1:1 |
| *Gfap*-C2 | Advanced Cell Diagnostics, 313211-C2 | 1:50 |
| *Il1b* | Advanced Cell Diagnostics, 316891 | 1:1 |
| *Olig2*-C2 | Advanced Cell Diagnostics, 447091-C2 | 1:50 |
| *Tmem119*-C3 | Advanced Cell Diagnostics, 472901-C3 | 1:50 |
