# Supplementary material for: Brain injury drives optic glioma formation through neuron-glia signaling
Source: Acta Neuropathol Commun. 2024 Feb 2;12:21. doi: 10.1186/s40478-024-01735-w (PMC10837936; doi:10.1186/s40478-024-01735-w)
Supplement: Supplementary file 2 — Additional file 2. Supplementary Figures. Fig. S1. Following unilateral optic nerve crush (u-ON-CR) performed at 6 weeks of age, the prechiasmatic region of Nf1flox/flox; hGFAP-Cre mice ipsilateral to the u-ON-CR exhibits (a) greater cellularity and (b) GFAP expression at 12 weeks of age. (c) Optic gliomas persist at 24 weeks of age in Nf1flox/flox; hGFAP-Cre mice following optic nerve crush performed at 6 weeks of age, as evidenced by increased (d) optic nerve volume and (e) proliferation (%Ki67+ cells) (n=5). (f) Following optic nerve crush (ON-CR) at 6 weeks of age, wild type (Nf1flox/flox) mice exhibit no change in (g) optic nerve volume (n = 5) or (h) proliferation (%Ki67+ cells; sham, n = 7; ON-CR, n = 6) when analyzed at 12 weeks of age. Scale bars: a, b Upper panel scale bar, 200 μm; lower panel scale bar, 50 μm d, g 100 μm; e, h 50µm. Two-tailed Student’s t test (ns, not significant). Asterisks denote the age at euthanasia and analysis. Fig. S2. Representative images of Nf1OPG mouse optic nerves following ON-CR at 6 weeks of age have increased (a) cellularity and (b) GFAP expression relative to the sham operation group. Optic nerves from Nf1f/R1809C; hGFAP-Cre mice following optic nerve crush at 6 weeks of age have increased (c) cellularity and (d) GFAP expression compared to the sham operation group when analyzed at 12 weeks of age. Scale bars: a-d upper panel scale bar, 200 μm; lower panel scale bar, 50 μm. Fig. S3. Following ON-CR at 6 weeks of age, RNAscope reveals increased numbers of (a) Tmem119+ cells and (b) Ccl5+ cells in the optic nerves of Nf1flox/flox; hGFAP-Cre mice at 12 weeks of age. PLX3397 (275mg/kg PLX) treatment reduces ON-CR-induced increases (c) in Blbp+ and Olig2+ cell content (%Blbp+, %Olig2+ cells; n=5), as well as (d) Ccl5 expression, in the optic nerves at 12 weeks of age relative to those fed the control (CTL) diet (n=3). (e) TAMs (Iba1+ cells) in 12-week-old Nf1flox/flox; hGFAP-Cre mice express p65-NFκB following ON-CR at 6 weeks [file 40478_2024_1735_MOESM2_ESM.docx]

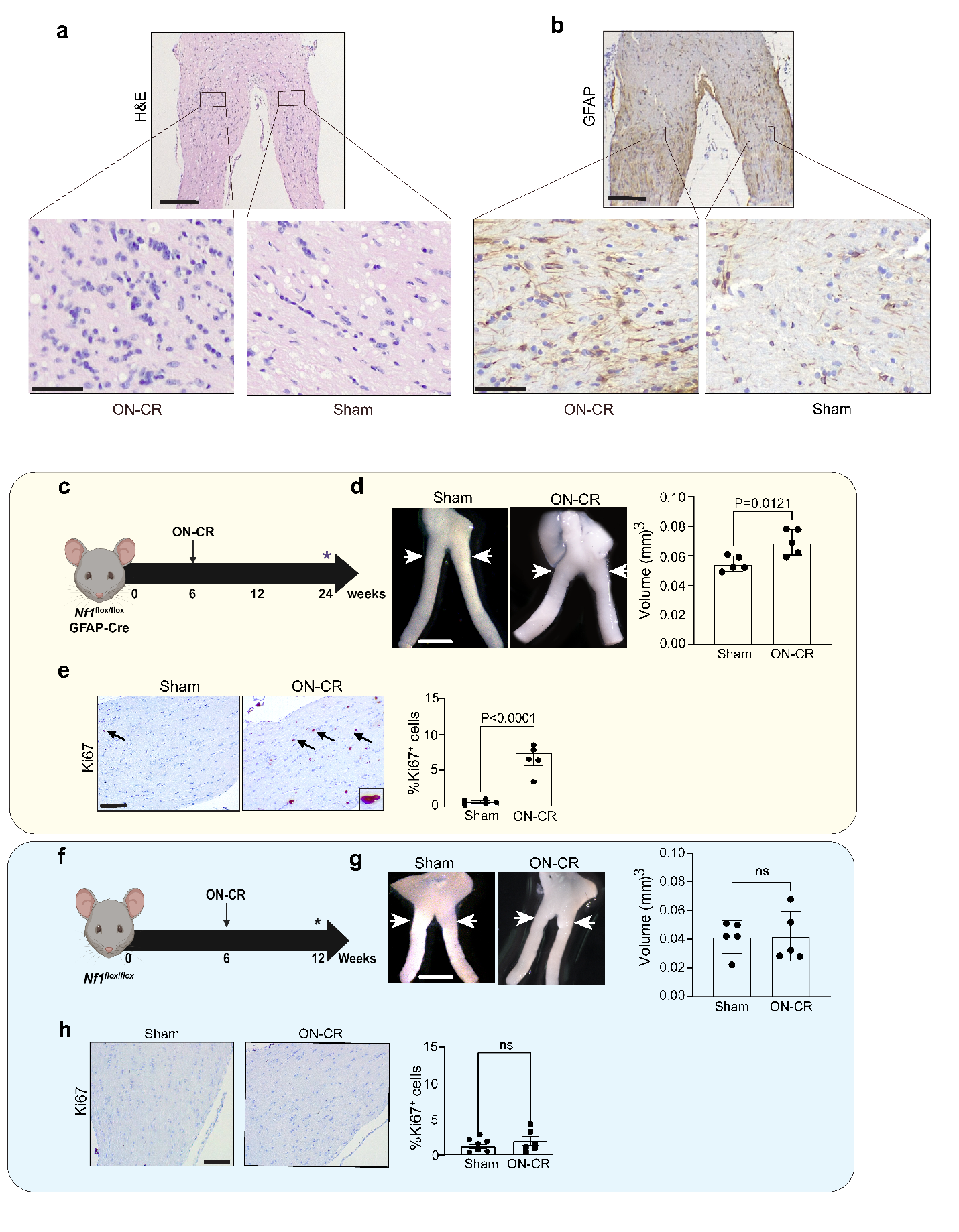


**Fig. S1.** Following unilateral optic nerve crush (u-ON-CR) performed at 6 weeks of age, the prechiasmatic region of *Nf1^flox/flox^*; hGFAP-Cre mice ipsilateral to the u-ON-CR exhibits **(a)** greater cellularity and **(b)** GFAP expression at 12 weeks of age. **(c)** Optic gliomas persist at 24 weeks of age in *Nf1^flox/flox^*; hGFAP-Cre mice following optic nerve crush performed at 6 weeks of age, as evidenced by increased (**d**) optic nerve volume and (**e**) proliferation (%Ki67^+^ cells) (*n*=5). (**f**) Following optic nerve crush (ON-CR) at 6 weeks of age, wild type (*Nf1*^flox/flox^) mice exhibit no change in **(g)** optic nerve volume (*n* = 5) or (**h**) proliferation (%Ki67^+^ cells; sham, *n* = 7; ON-CR, *n* = 6) when analyzed at 12 weeks of age. Scale bars: **a, b** Upper panel scale bar, 200 μm; lower panel scale bar, 50 μm **d, g** 100 μm; **e, h** 50µm. Two-tailed Student’s *t* test (ns, not significant). Asterisks denote the age at euthanasia and analysis.


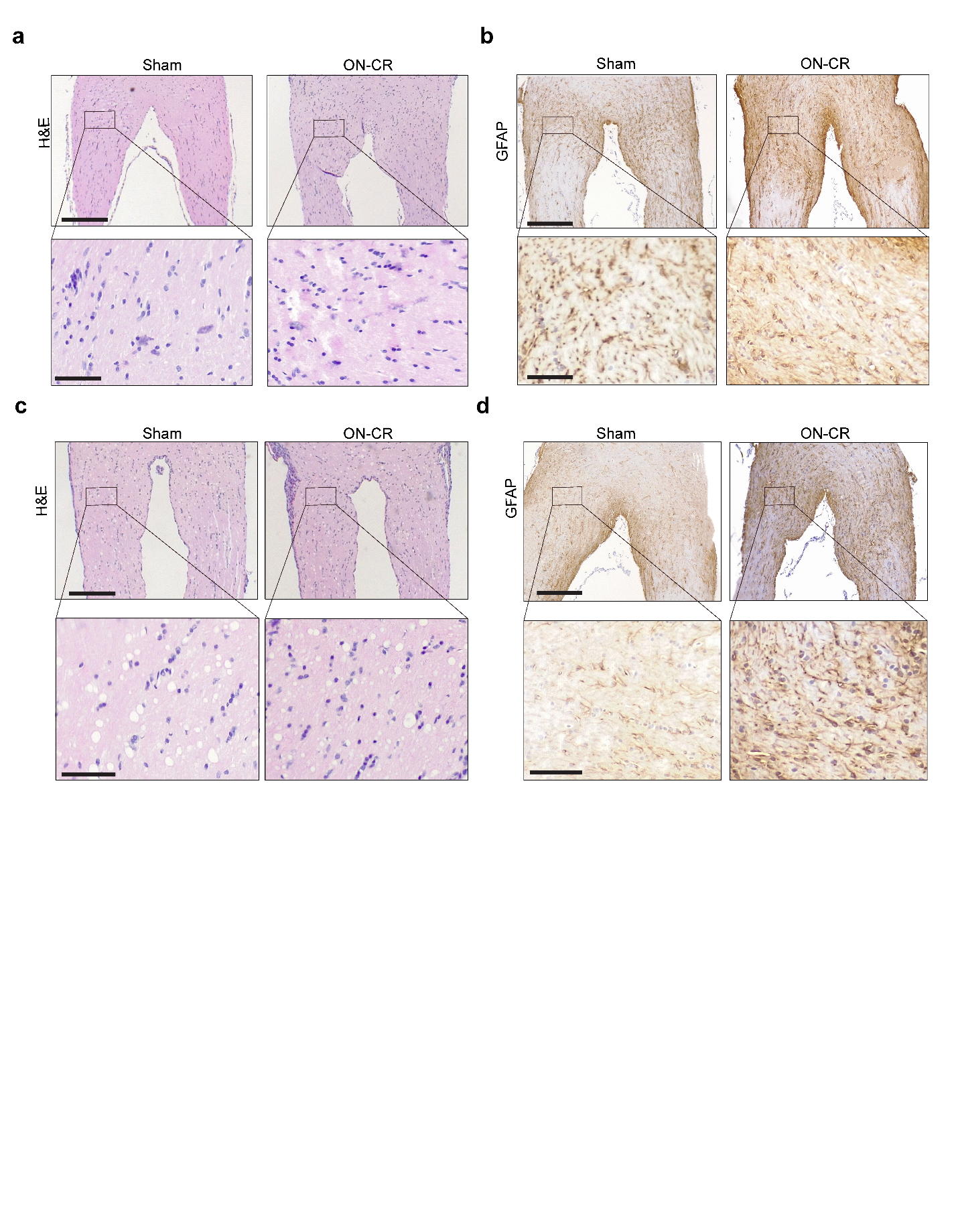


**Fig. S2.** Representative images of *Nf1*^OPG^ mouse optic nerves following ON-CR at 6 weeks of age have increased **(a)** cellularity and **(b)** GFAP expression relative to the sham operation group. Optic nerves from *Nf1*^f/R1809C^; hGFAP-Cre mice following optic nerve crush at 6 weeks of age have increased **(c)** cellularity and **(d)** GFAP expression compared to the sham operation group when analyzed at 12 weeks of age. Scale bars: **a-d** upper panel scale bar, 200 μm; lower panel scale bar, 50 μm.


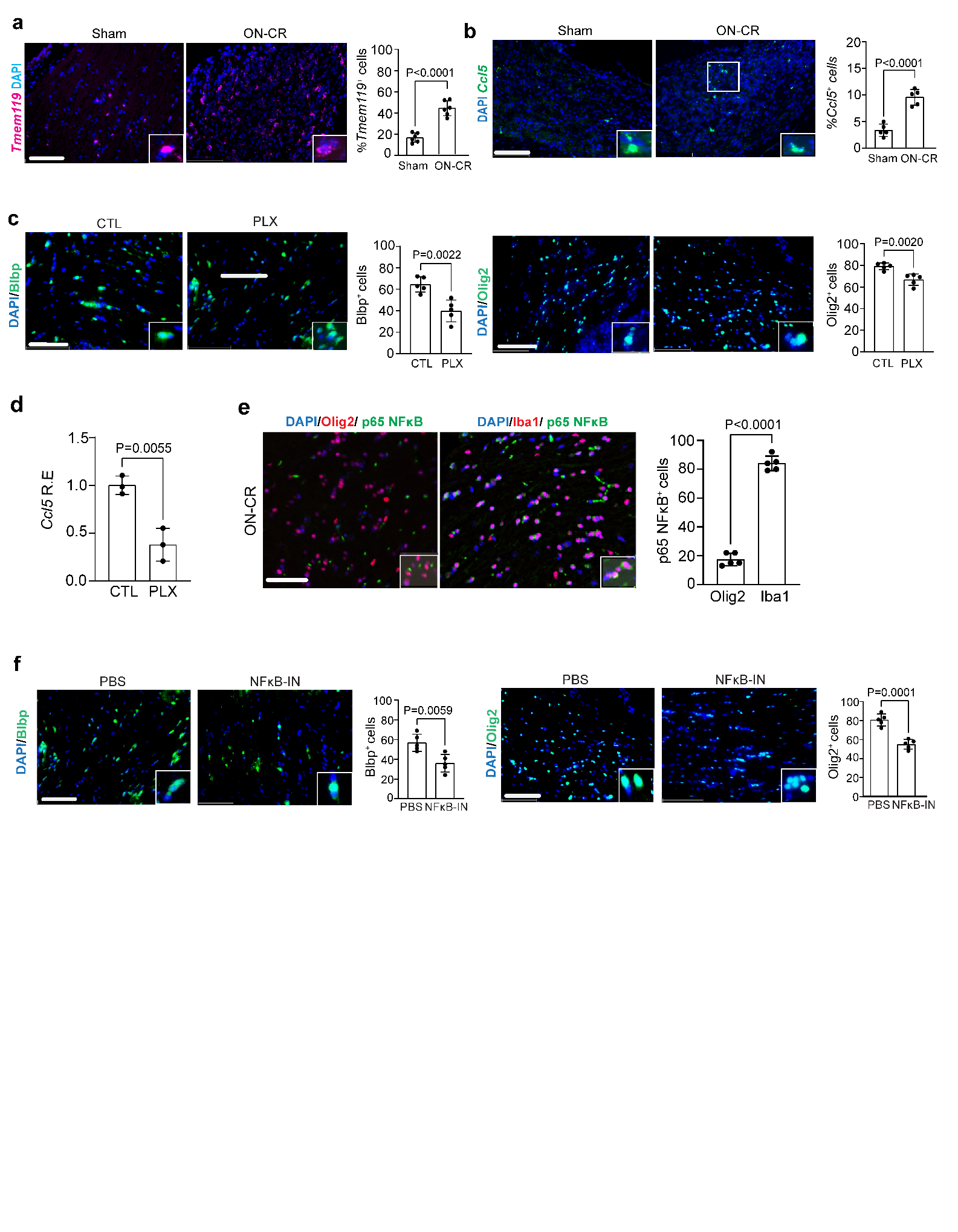


**Fig. S3.** Following ON-CR at 6 weeks of age, RNAscope reveals increased numbers of (**a**) *Tmem119^+^* cells and (**b**) *Ccl5*^+^ cells in the optic nerves of *Nf1^flox/flox^*; hGFAP-Cre mice at 12 weeks of age. PLX3397 (275mg/kg PLX) treatment reduces ON-CR-induced increases **(c)** in Blbp^+^ and Olig2^+^ cell content (%Blbp^+^, %Olig2^+^ cells; *n*=5), as well as **(d)** *Ccl5* expression, in the optic nerves at 12 weeks of age relative to those fed the control (CTL) diet (*n*=3). **(e)** TAMs (Iba1^+^ cells) in 12-week-old *Nf1^flox/flox^*; hGFAP-Cre mice express p65-NFκB following ON-CR at 6 weeks of age. *Nf1^flox/flox^*; hGFAP-Cre mice that underwent ON-CR at 6 weeks of age exhibit reduced Blbp^+^ and Olig2^+^ (%Blbp^+^, %Olig2^+^ cells; *n*=5) cell content at 12 weeks of age following **(f)** Caffeic acid phenethyl (10mg/kg CAPE, NFκB inhibitor, NFκB-IN) treatment relative to PBS-treated controls. Data are presented as the means ± SEM. Scale bars: **a, b, e, f, g** 50 μm; Two-tailed Student’s *t* test.


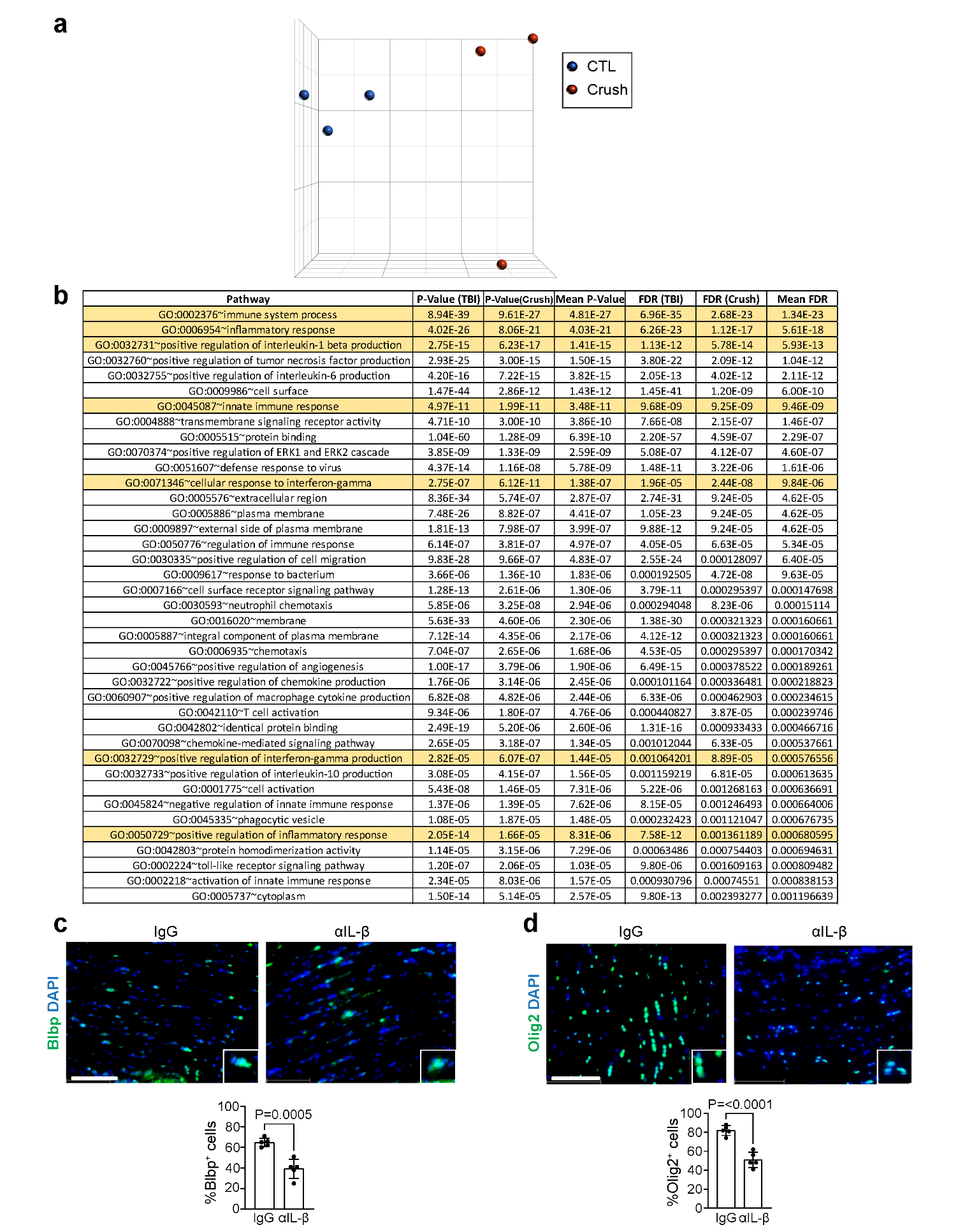


**Fig. S4.** (**a**) Analysis of differential gene expression by bulk RNA sequencing of optic nerves from 12-week-old *Nf1^flox/flox^*; hGFAP-Cre mice following optic nerve crush (ON-CR; n=3) or sham surgery (n=3) at 6 weeks of age. (**b**) R studio version 2023.03.0 was used to identify enriched pathways and to calculate the mean and p-value for ON-CR relative to sham mouse groups, as well as to determine the FDR for each shared pathway. The 40 most significant pathways were identified using the mean FDR ≤ 0.05, and log fold changes greater or equal to ±5. **(c, d)** αIL1β treatment after ON-CR at 6 weeks of age results in reduced optic nerve Blbp^+^ and Olig2^+^ cell content in *Nf1^flox/flox^*; hGFAP-Cre mice at 12 weeks of age compared to IgG controls (%Blbp^+^, %Olig2^+^ cells; *n*=5). Data are presented as the means ± SEM. Scale bars: **c, d** 50μm; Two-tailed Student’s *t* test.


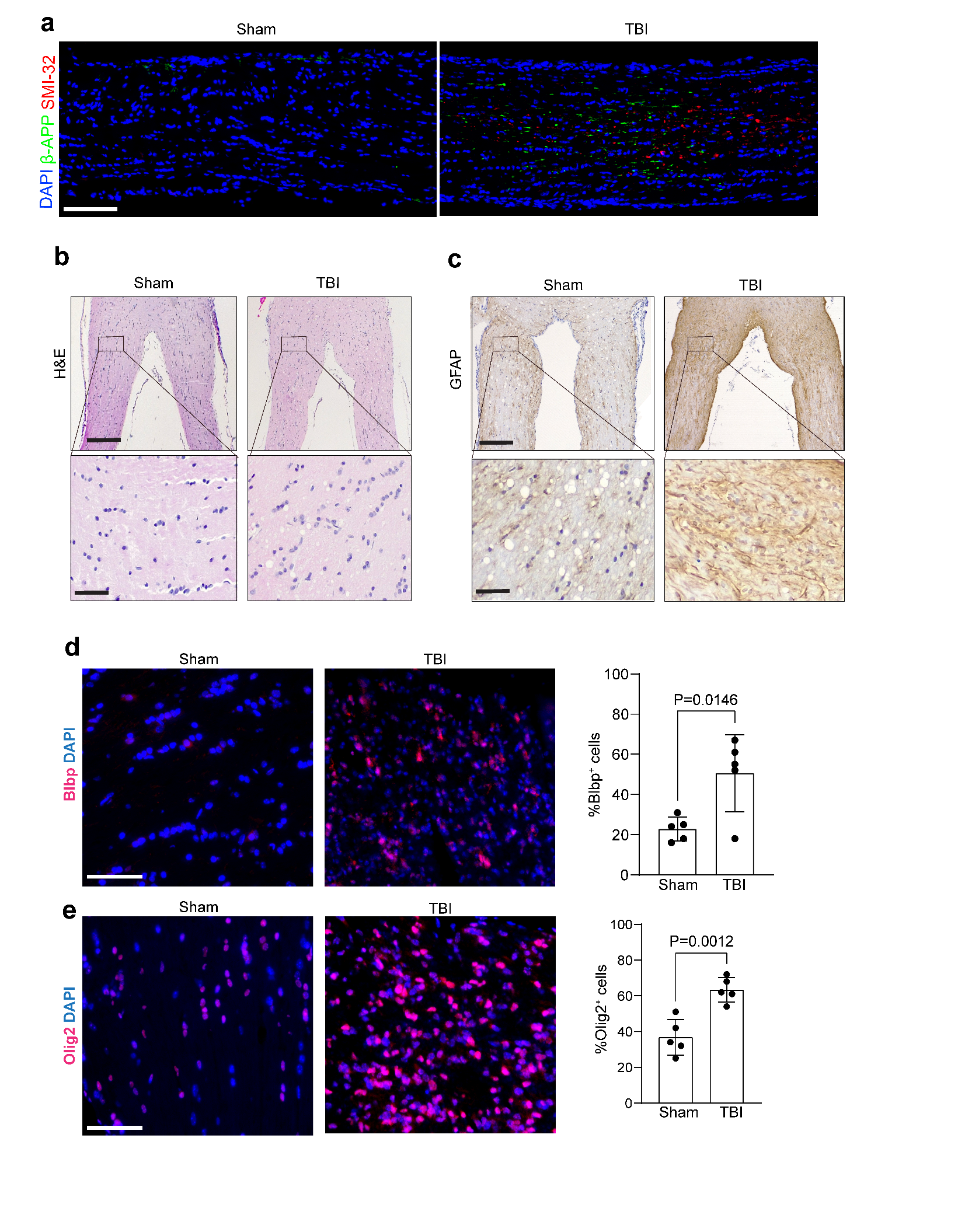


**Fig. S5.** (**a**) Increased β-APP and SMI-32 expression is seen in the optic nerves of *Nf1^flox/flox^*; hGFAP-Cre mice 7 days after TBI (modCHIMERA). Following TBI at 6 weeks of age, increased (**b**) optic nerve cellularity and (**c**) GFAP expression is observed by immunohistochemistry in *Nf1^flox/flox^*; hGFAP-Cre mice at 12 weeks of age relative to sham controls. Immunofluorescence reveals increased (**d**) %Blbp^+^ (*n* = 5) and (**e**) %Olig2^+^ (*n* = 5) cells in *Nf1^flox/flox^*; hGFAP-Cre mice at 3 months of age following TBI at 6 weeks of age compared to sham injury controls. Data are presented as the means ± SEM. Scale bars: **a** 50 μm, **b, c** upper panel bar, 200 μm; lower panel scale bar, 50 μm, **d, e** 40μm. Two-tailed Student’s *t* test.
